# Responding to Distress Choosing Between Care and Food: Attachment Orientation and Emotion Regulation

**DOI:** 10.3389/fpsyg.2022.930168

**Published:** 2022-07-20

**Authors:** Arcangelo Uccula, Mauro Enna, Claudio Mulatti

**Affiliations:** ^1^Department of History, Human Sciences and Education, University of Sassari, Sassari, Italy; ^2^Department of Psychology and Cognitive Science, University of Trento, Trento, Italy; ^3^Department of Developmental Psychology and Socialisation, University of Padova, Padova, Italy

**Keywords:** seek social support, comfort, anxiety, threat, avoidance

## Abstract

According to attachment theory, care-seeking is the primary coping strategy in threatening situations. However, anxious and avoidant individuals often use secondary regulation strategies. The purpose of this study was to test whether, in a potentially threatening situation, the participants' attachment orientation affects whether they prefer to resort to care or food to regulate their negative emotions. Ninety-two participants took part in an experimental situation in which they had to choose between pictures of care or food, following the presentation of threatening images randomly alternating with neutral ones. Results showed that care pictures were chosen to a greater extent in the threatening condition compared to the food pictures and the neutral condition, without distinction of attachment orientation. In addition, in threatening condition, anxious individuals chose to care less than non-anxious individuals. Finally, avoidant participants chose care pictures to a lesser extent than individuals low on avoidance in the neutral condition, but not in the threatening condition. In conclusion, attachment anxiety was associated with more difficulty in the choice of representation of care in a threatening condition, while avoidant individuals show their defensive strategies in the neutral condition rather than in the threatening condition.

## Introduction

Within the classical framework of the attachment theory, when people experience a threatening situation, they activate the attachment system and tend to seek the proximity of significant others (Bowlby, 1969). Support-seeking, in fact, plays a critical role in decreasing the impact of psychophysiological stress (Coan et al., 2017; Feeney and Collins, 2019). Later studies have shown that this strategy in dealing with a threatening stimulus begins to be shaped during a person's experience in early childhood (Waters and Waters, 2006). Of particular, relevance is the behavioral pattern of attachment that an individual has built during infancy through the interactions with the caregiver(s): different attachment styles can either facilitate or interfere with the search for proximity as a strategy to deal with the emotions triggered by a given threat (Waters and Waters, 2006). Whether to seek the proximity of others or not is, thus, an individual difference, which reflects the strategy that individuals use to regulate their emotions, a strategy that results from the individual's pattern of attachment (Mikulincer and Shaver, 2019).

Adults' attachment orientation—shaped in childhood through the interaction with the caregiver (Ainsworth et al., 1978)—can be organized along two emotional dimensions: anxiety and avoidance (Bartholomew and Horowitz, 1991; Mikulincer and Shaver, 2019). Within this two-dimensional framework, different attachment orientations are paired with different emotion regulation strategies, stress coping strategies, and the individuals' expectations concerning their capability to deal with threatening situations (Caldwell and Shaver, 2012). Anxious individuals find it difficult to seek support, but adopt indirect strategies that intensify the expression of emotions to attract the attention of significant others (Mikulincer and Shaver, 2016). Conversely, avoidant individuals downregulate threat-related emotions with strategies that deny stress or divert attention from the source of the emotions. Thus, attachment theory provides a powerful framework for clustering how individuals tend to regulate their emotions (Brumariu, 2015; Mikulincer and Shaver, 2019). Although, during infancy, the primary strategy for restoring the emotional balance following a potential threat is seeking the proximity of significant others (Bowlby, 1969); in adulthood, this strategy is not necessarily the behavior of seeking physical proximity, but it may also consist in the activation of mental representations of their symbolic presence (Mikulincer and Shaver, 2016).

This primary strategy, however, is more easily available to those individuals who have had a positive experience in childhood with their caregivers' availability and have, thus, developed an attachment style characterized by both the low avoidance and low anxiety (Waters and Waters, 2006): those individuals tend to seek support to regulate their negative emotions in times of need. On the contrary, individuals either high in anxiety or high in avoidance have not had a positive experience with the availability of their caregiver. Those individuals have learned that they cannot rely on others when they feel uneasy and, thus, have to resort to other strategies to regulate their emotions in case of need (Mikulincer and Shaver, 2019).

It has been shown that some individuals prefer resorting to food intake to regulate their negative emotions (typically anxiety) when they experience distress (Evers et al., 2018; Devonport et al., 2019). In other studies, the presentation of anxiogenic (vs. secure) prime stimuli increased snack consumption (Wilkinson et al., 2013). So, the negative emotions triggered by distress might be regulated by the search for proximity by some individuals and by food intake by others. In accordance with these premises, our study aimed to test whether attachment orientation affects the way they respond to a perceived potential threat to cope with their negative emotions.

Receiving adequate parental care and food in childhood is essential to promote healthy physical and mental development (Atzil et al., 2018). In addition, food can perform a regulatory function during affective development. In fact, in addition to having a nutritional function, food also plays an interpersonal regulatory role, for example, when parents use food to soothe their children's negative emotions or discomfort (Stifter et al., 2011; Hamburg et al., 2014). Studies show that mothers' attachment anxiety is associated with using emotional feeding strategies with their children with the aim of feeling closer to them (Hardman et al., 2016). Moreover, parents–child concordance in the child's eating behavior is lower in families where the child shows an insecure attachment with respect to families where the child shows a secure attachment (Uccula et al., 2012). Food consumption can, in fact, activate cognitions linked to the relationship through a learned empathic symbolization of eating experiences within close relationships (Hamburg et al., 2014). For example, comfort food consumption was associated with positive social interactions and a reduction in feelings of loneliness (Troisi and Gabriel, 2011). Consequently, a link between emotion regulation, food intake, and attachment style has been found (Faber et al., 2018) and eating disorder symptoms have been associated with avoidance in intimacy and abandonment-related anxiety (Gonçalves et al., 2019). Thus, to regulate their negative emotions, individuals with anxious attachment might attempt to seek support through the intensification of their emotional expression—a strategy with limited coping efficacy—and might resort to food intake as coping strategy. This happens because individuals with anxious attachment have ambivalent cognitions regarding the context of care-seeking: in case of need, while seeking support for obtaining care and security, they also experience doubts regarding the actual availability of care and support (Vogel and Wei, 2005). Instead, avoidant attachment individuals attempt to block threat-related emotions in stressful situations by adopting a defensive behavior, attentional disengagement (Mellor and Psouni, 2021), denying stress, and, in addition, they do not typically seek the support of others (McLeod et al., 2020). Although avoidant individuals' difficulties in seeking support were found, several studies have shown that such difficulties can be reduced in particular conditions (Rholes et al., 2021). It has been shown that the undeniable, self-evident availability of significant others decreases the frequency of occurrence of defensive behaviors in adult avoidant individuals (Overall et al., 2022). In addition, Diamond and Fagundes (2010) reported a discrepancy between the self-reported dismissive feelings and the measures of physiological activation of individuals with avoidant attachment in a stressful context (e.g., the exposure to infant crying; Ablow et al., 2013). It was as if their defensive attitude was a strategy to mask (i.e., to cope by denying) their emotions, as indexed by their physiological activity. Other studies confirm that avoidant defenses collapse under pressure (Mikulincer and Shaver, 2016).

### Present Study

In accordance with these premises, the purposes of the study were: (a) to test whether the representations of care or food were associated with threatening vs. neutral situations and (b) to test whether the attachment orientation of a given individual affects the way they react to a perceived potential threat. More in detail, the following hypotheses were put forward:

- First, regardless of the attachment orientation, we expected that participants in the threatening condition would choose representations of care more often than food ones and with respect to the neutral condition.
- Second, in the threatening condition, we expected that individuals with an anxious attachment would choose representations of care less often than low-anxious individuals and small-to-no differences in the frequency of choice of representations of care between avoidant and low-avoidant individuals.
- Third, in the neutral condition, we expected less frequently chosen representations of care by avoidant individuals and small-to-no differences in anxious attachment individuals.

## Materials and Methods

### Participants

Ninety-two Italian students volunteered to take part in this study (48 women, 44 men, *M*_age_ = 22.57 years, *SD* = 2.87; age range: 19–31 years). A priori power analysis was conducted using G^*^Power version 3.1.9.7 (Faul et al., 2007) to determine the minimum sample size required to test the study hypothesis. Results indicated the required sample size to achieve 85% power for detecting a small/medium effect (0.3; Cohen, 1988), at a significance criterion of α =0.05, was *N* = 82 for a within-participant design. Thus, the obtained sample size of *N* = 92 is adequate to test the study hypothesis. The experimental protocol conformed to the Declaration of Helsinki and was approved by the Ethics Committee of the University of Padova, Italy. Written informed consent was obtained from all the participants.

### Design and Statistics

The differences between care and food choices [here operationalized as a preference for pictures representing a caring scenario, Rowe et al. (2020) or as a preference for pictures representing various kinds of comfort food] in the threatening and neutral conditions were analyzed using a within-subject ANOVA. *Post-hoc* pairwise comparisons were done, if the omnibus test found a significant difference. After that, the dimensions of anxiety and avoidance of attachment were included in the analysis as covariates. In order to highlight possible significant associations, we will show the differences in the choices by using standardized *Z*-scores (−1 *SD* vs. 1 *SD*) of attachment orientations.

The potential confounding variables that would be correlated with the choices of food were considered. Some studies have found an association between restrained eating (Evers et al., 2018), body mass index (BMI), and eating behavior (Wilkinson et al., 2018). Likewise, the status of hunger at the time of the experiment was evaluated. However, in experimental studies (food-related attentional bias), these associations were sometimes not found (Hardman et al., 2021); therefore, we wanted to test whether these variables were associated with food image choice. Before the main analyses, backward stepwise regressions between BMI, restrained eating (yes or no), the status of hunger (1 = Not at all hungry; 5 = Extremely hungry), and food choice were performed. The variables that showed no significant association (*p* > 0.05) were not included in further analysis. Statistical analysis was conducted using SPSS software version 26.

### Materials

The attachment style was assessed with the Italian version (Busonera et al., 2014) of the Experiences in Close Relationships-Revised (ECR-R; Fraley et al., 2000). The ECR-R is a widely used self-report questionnaire to assess the two basic dimensions of anxiety and avoidance of attachment and has excellent internal consistency (Fraley et al., 2000). In the present study, Cronbach's alpha coefficients were 0.91 for the anxiety items and 0.89 for the avoidance items. The means of the ECR-R were in line with the Italian adaptation.

The stimuli were 180 pictures selected from various sources:

- Forty pictures from the International Affective Picture System (IAPS; Lang et al., 2008)^1^ were used for the threatening (e.g., lunging dogs) and neutral (e.g., domestic objects) conditions. Mean ratings of valence and arousal were as per by Lang et al., 2008 (nine points Likert-type scale: 1 = negative, low). Valence and arousal were significantly different between the two conditions, *F*_(1, 38)_ = 264 and *F*_(1, 38)_ = 438, respectively. The threatening images we used had also been used by Kappenman et al. (2015) and showed that they were capable of activating the threatening system. Table 1 shows the descriptive statistics of the ECR-R questionnaire and the IAPS pictures.
- Forty pictures from the Food-Pics_Extended (Blechert et al., 2019)^2^ were selected. The pictures were of typical comfort foods (sweet and salty snacks) and they were randomly divided into two lists of 20 items: one for the neutral condition and one for the threatening condition. Mean ratings of calories, palatability, and craving were as per by Blechert et al. (2019): the two lists did not significantly differ on any dimension, all *Fs* < 1.
- Forty pictures from the Besançon Affective Picture Set-Adult (BAPS-Adult; Szymanska et al., 2019)^3^ were selected. The 40 pictures depicted comfort-related scenarios where care was represented (e.g., an adult comforts an infant in distress). Two lists of 20 pictures were created: one for the neutral condition and one for the threatening condition. Mean ratings of valence, arousal, and perceived comfort were as per by Szymanska et al. (2019): the two lists did not significantly differ on any dimension, all *F*s < 1.
- Sixty neutral pictures from the IAPS and the Food-Pics_Extended were selected for the 20 filler trials.

**Table 1:** Mean and SD of attachment orientations and prime stimulus in the two conditions.

	**ECR-R**		**IAPS threatening**		**IAPS neutral**	
	**Anxiety**	**Avoidance**	**Valence**	**Arousal**	**Valence**	**Arousal**
*M*	3.30	2.45	2.52	6.62	5.11	2.91
*S.D*.	0.88	0.73	0.64	0.41	0.32	0.68

### Procedure

Demographics include participants' age and gender. Body mass index (BMI) and restrained eating status were collected, as well as a rating of the current hunger level prior to the experiment. Participants filled in the ECR-R questionnaire and performed the experiment in a counterbalanced way: half of them started with the questionnaire and the other half started with the experiment. A program coded within the OpenSesame software (Mathôt et al., 2012) controlled the presentation of the stimuli and the recording of the response. Stimuli were displayed on a 15.6 inches monitor, set at a distance of 60 cm from the participant. Before initiating the experiment, participants were presented with the following instructions (instructions were presented in Italian, below is the English equivalent):

“You will see neutral images and other images that will probably make you feel negative emotions, then you will have to choose one of the two images that follow, the one that at that time can help you to overcome the negative emotion of the single image you saw before.”

Then, the experiment started and consisted of 60 randomly intermixed trials: 20 trials in the threatening condition, 20 trials in the neutral condition, and 20 filler trials. Each trial began with a fixation dot presented for 500 ms in the center of the screen; at its offset, a prime picture replaced the fixation dot and remained visible for 3 s. In the neutral condition, each prime picture had a neutral content. In the threatening condition, each prime picture had threatening content. In the filler trials, the prime picture had a neutral content. At the offset of the prime picture, two probe pictures were concurrently presented side-by-side. In the neutral and threatening conditions, one of the two pictures depicted a caring scenario, whereas the other picture depicted food. In the filler trials, the two probe pictures had a neutral content. Participants had to choose at their own discretion the picture they favored the most between those two probe pictures. The two probe pictures remained visible until participants responded. The relative position of the two pictures (left vs. right) was counterbalanced (Uccula et al., 2020b). The 60 experimental trials were preceded by 10 practice trials.

## Results

To evaluate our hypotheses, we calculated the relative frequency of choice of care vs. food pictures. Table 2 shows the means and SDs of the choices according to conditions.

**Table 2:** Means, SD, and differences between care and food choices in the two conditions.

	**Neutral**	**Threatening**	**Differences**
	***M (SD)***	***M (SD)***	**Neutral—threatening**
Care	9.89 (5.03)	16.15 (3.86)	−6.26
Food	10.11 (5.03)	3.85 (3.86)	6.26
Differences care—food	−0.22	12.30	

From the stepwise regression to selection for potential confounding variables, none of those evaluated: BMI (*r* = 0.082, *p* = 0.218), restrained eating status (*r* = −0.011, *p* = 0.458), and hunger (*r* = −0.100, *p* = 0.172) were associated with the choices of food in neutral and threatening conditions. Table 3 shows the descriptive statistics of the potential confounding variables.

**Table 3:** Descriptive statistics of the confounding variables.

	***N***	***%***	***M (SD)***
BMI			21.83 (3.46)
**Restrained eating**			
No	59	64.1	
Yes	33	35.9	
**Hunger**			
Not at all hungry	36	39.1	
Lightly hungry	42	45.7	
Moderately hungry	13	14.1	
Very hungry	1	1.1	

**Figure 1:**
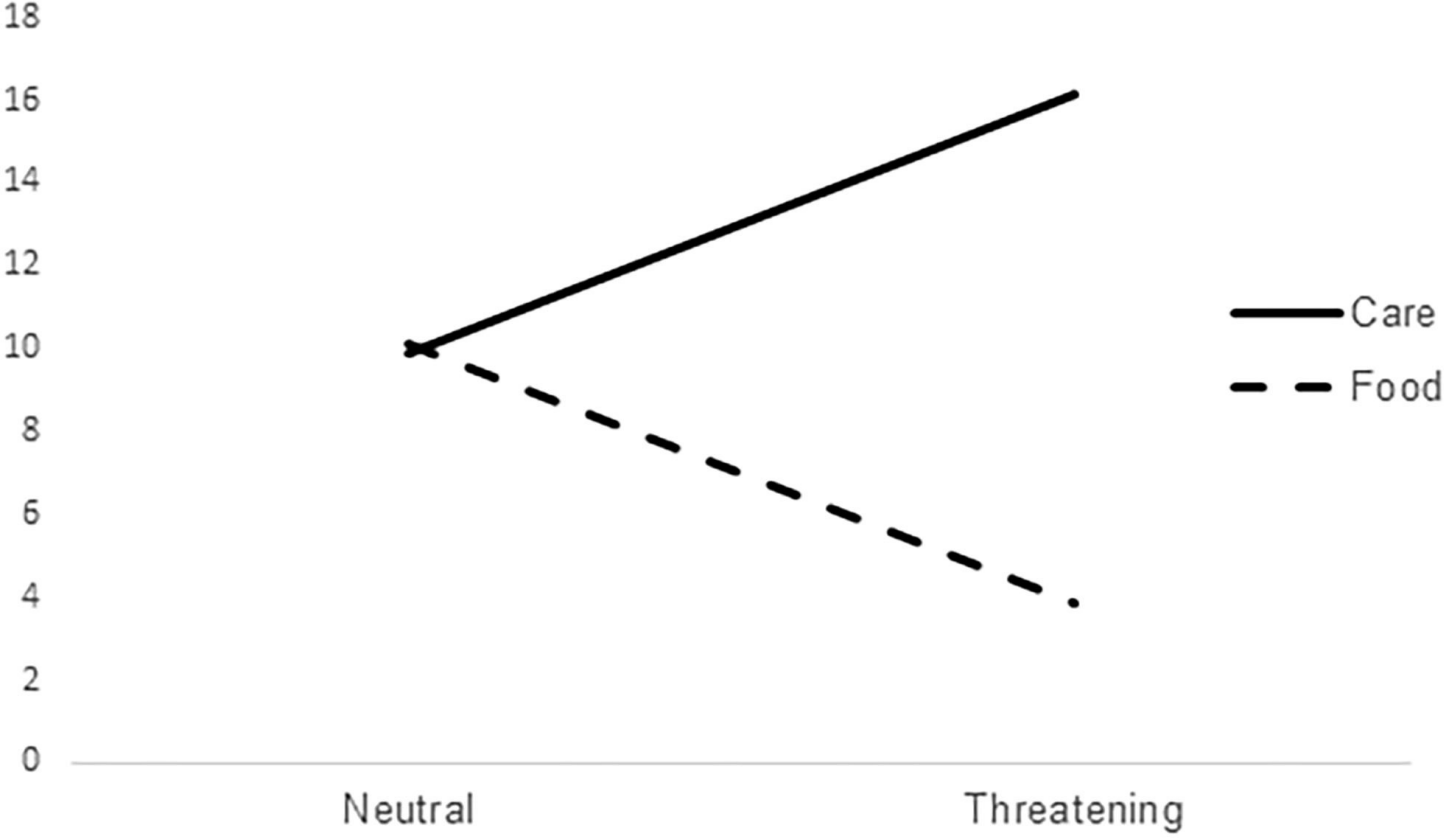
Means of care and food choices in the two conditions.

In order to verify our first hypothesis, the repeated measures ANOVA was carried out. The first hypothesis was that participants choose care pictures more often than food pictures in the threatening condition and that participants choose care pictures more often in the threatening condition than the neutral condition, regardless of the attachment orientation. Results of the omnibus ANOVA indicated a significant effect, *F*_(1, 91)_ = 149.867, *p* < 0.001. Because the choice of food vs. care is mutually exclusive, this result concerns both the predictions of the first hypothesis. Out of the 20 trials of the neutral condition, care pictures were selected 9.89 times on average (*SD* = 5.03), i.e., almost half of the time. Out of the 20 trials of the threatening condition, care pictures were selected 16.15 times on average (*SD* = 3.86) vs. a mean of 3.85 for food choices (Figure 1).

The *post-hoc* comparisons (Bonferroni correction) showed that in the threatening condition, the differences between care and food choices were significantly greater than in the neutral condition [mean difference – 0.22 + 12.30 = 12.52, *t* = 12.24, *p* < 0.001, 95% *CI* (10.49, 14.55)].

The repeated measures ANCOVA with the dimensions of anxiety and avoidance of attachment as covariates were performed. The results showed a main effect, *F*_(1, 89)_ = 5.298, *p* = 0.024, and interactions effects between care and food choices with both the attachment anxiety, *F*_(1, 89)_ = 4.819, *p* = 0.031 and attachment avoidance, *F*_(1, 89)_ = 9.243, *p* = 0.003.

**Figure 2:**
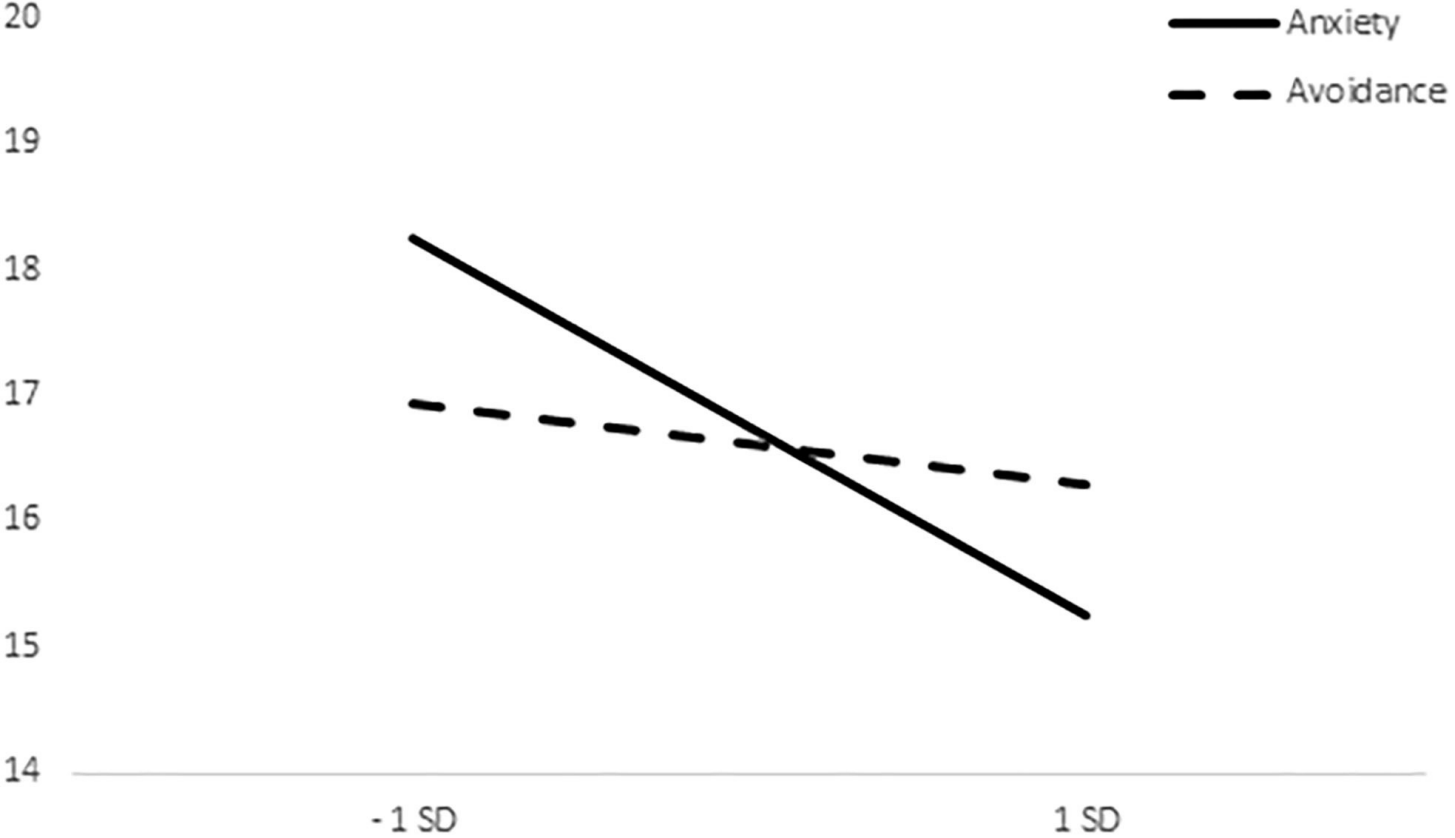
Differences between means of care pictures choices in the threatening condition.

**Figure 3:**
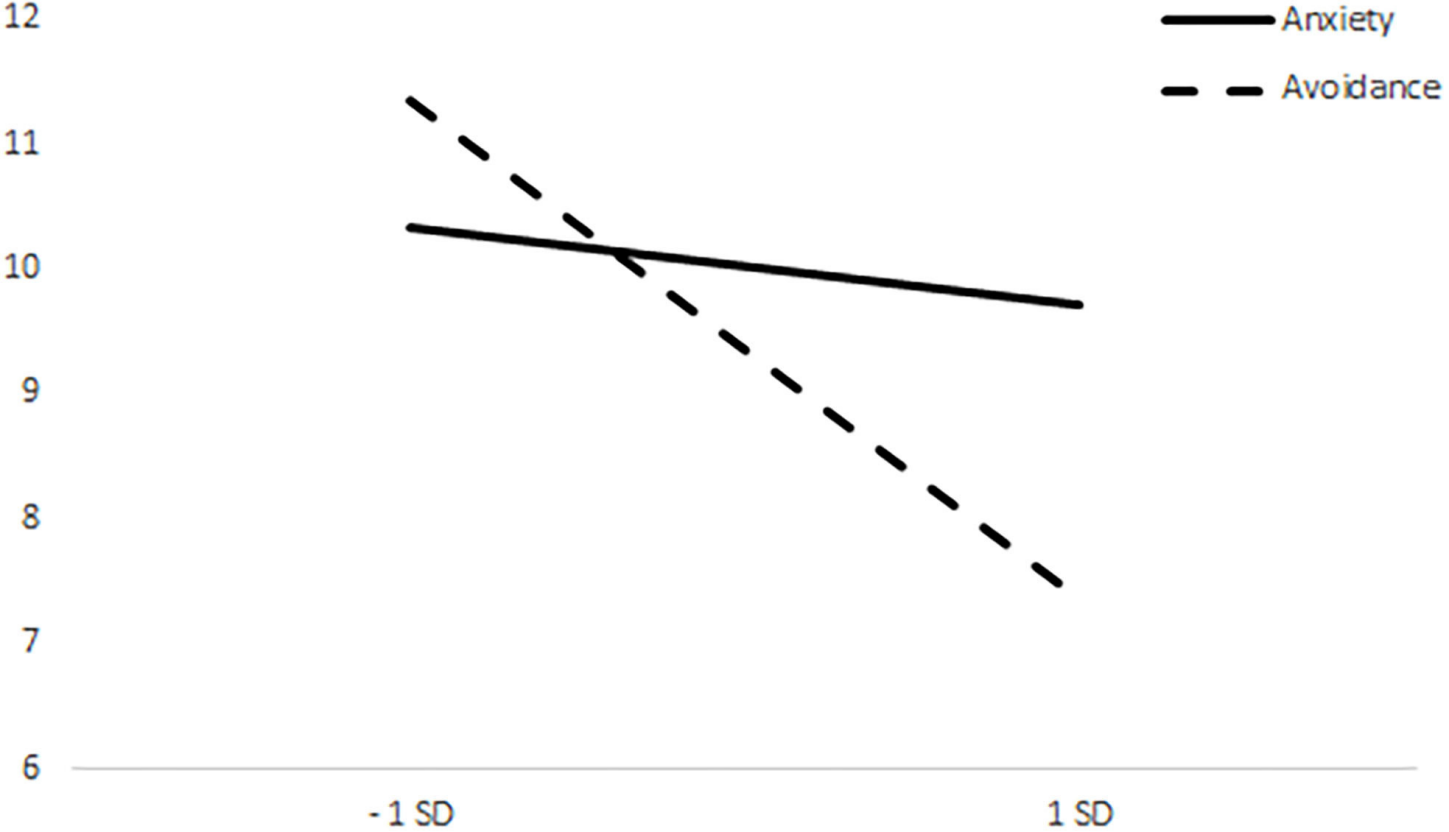
Differences between means of care pictures choices in the neutral condition.

Whereas the effect of anxiety is significant in the threatening condition, *t* = −3.042, *p* < 0.005, it is not significant in the neutral condition, |t| < 1 (Figure 2). In addition, whereas the effect of avoidance is not significant in the threatening condition, |t| < 1, it proves significant in the neutral condition, *t* = −2.650, *p* < 0.01 (Figure 3).

## Discussion

The purposes of the present study were to test whether—in a threatening (vs. neutral) condition—participants resort to care more than to food pictures to regulate their emotions and to test whether this tendency is associated with attachment orientation. Our finding accords with the attachment theory framework: under threat, individuals seek the support of others to regulate their emotional distress. This finding confirms, even in an experimental situation, that the attachment-related mental representations of care and social support are activated and are associated with a greater choice of care pictures, following the experience of an emotional distress/threat. On the other hand, it should be noted that in the neutral condition, in the absence of distress, the choices of care are almost equivalent to the food pictures choices.

However, our findings also show that these choices are associated with the individual's attachment orientation. In the threatening condition, individuals with an anxious attachment choose care pictures less often than those individuals who have a low-anxious attachment. This result is consistent with the ambivalent representation of attachment characterizing this attachment style: their mental representations of the attachment figure are unstable and unreliable; the attachment figure is inconstant, it might be present or it might not be present (Cheng et al., 2015). The intensification of the expression of emotions as a signal for inconstant caregivers/partners together with negative expectations regarding their availability and their support (Gökdag, 2021) might lead anxious individuals to attempt to regulate their emotions through food (Wilkinson et al., 2018) instead of through social support. In fact, individuals with a higher level of anxious attachment could have a negative image of themselves as unlovable and could develop an attitude of distrust toward others (Santona et al., 2022). In our study, the relative higher preference for food pictures in anxious individuals is also in line with clinical studies showing a greater risk (Pace et al., 2022) and incidence of eating disorders in the anxious attachment (Tasca and Balfour, 2014).

Conversely, in our findings regarding the threatening condition, avoidant individuals similarly choose care and food as the non-avoidant individuals. Many studies show that individuals with avoidant attachment tend not to seek social support, but adopt a defensive coping strategy following exposure to threatening events and disengaging from others (Mikulincer and Shaver, 2016): this despite their physiological reaction and emotional response to the event is present (Diamond and Fagundes, 2010). However, when the intimacy of a situation is manageable and prorelationship, then, if the presence and availability of the support are undeniable, they can give up their dominant defensive strategy and resort to seeking social support in case of need (Stanton et al., 2017). Other studies confirm that individuals with an avoidant attachment can contrast their default defensive response and express their need for social support more openly (Overall et al., 2022). The absence of differences in the choice of care pictures in the threatening condition that we found is consistent with this view: the pictures depicting a care scenario of an adult–child relation (a strong symbolic representation of care) can activate the attachment-related mental script of the support availability and allows the avoidant individuals to choose carefully as the non-avoidant individuals.

Conversely, in a more neutral condition, avoidant individuals are less motivated to choose care, they do not have to cope with negative emotions, and they resort to their default defensive behavior, which, in our experiment, is indexed by the lower frequency of care choice shown by the avoidant as opposed to the non-avoidant. This is congruent with the view that the “neutral,” default mode of avoidant individuals, entails the mental representation of the unavailability of the caregivers and that their attachment system was preconsciously deactivated (Mikulincer and Shaver, 2016). The experimental environment in which the experiment was carried out might have offered a set of conditions that favored the plasticity of avoidant behavior, thus allowing these individuals to differentiate their responses between the two levels of emotional context.

From a developmental perspective, these findings obtained with adults are partially consistent with previous results with children (Uccula et al., 2020a), although caution must be used in comparing categorical assessment of attachment during childhood with dimensional assessment in adulthood. The behavior of anxious and secure children (low anxiety and low avoidance in adults) in the threatening condition is similar. However, unlike adults, in avoidant children, a greater propensity to choose food in the threatening condition has been observed. This result could be interpreted by the different regulatory mechanisms underlying avoidant defenses in the two age groups, which may affect the emotional impact of the threat (e.g., to inhibit emotional states) and/or the choice of care (e.g., interference with the mental representation of support seeking).

In summary, our results clearly show that in the threatening situation, participants prefer representations of care as an emotional regulatory strategy. Within this general propensity, our results confirm that individuals with high (vs. low) attachment anxiety tend to choose secondary regulatory strategies, such as food. On the other hand, the choices of avoidant individuals in the threatening condition seem inconsistent with what is typically observed in these cases. Usually, in fact, avoidant individuals tend to not select representations of intimacy as a regulatory strategy, both in real-life contexts and in simulated contexts, and instead prefer secondary regulatory strategies in stressful situations. However, these defenses in our study emerge in the neutral condition, whereas in the threatening condition, it shows, also in line with recent studies, that in some circumstances when avoidantly attached individuals are distressed, they cannot use their normal defenses to regulate their emotions (Girme et al., 2015). The avoidant orientation in this study also emerges as rich insights for future research aimed at better defining the characteristics of this style, which has not yet found an agreed upon definition among researchers.

A potential limit has to do with the technique we employed to induce threat, since it might have limited ecological validity. However, the IAPS has been validated by a multiplicity of studies and has become a research paradigm and their emotional effects are well-established in the literature. Similarly, the constrained choice between pictures of food and pictures of a caring scenario, although they concern two strong forms of reward (Pool et al., 2016) and emotional regulation (Faber et al., 2018), might be considered as a limit of our study that will need to be addressed in future studies. A further limitation is that the pictures we used to prime the threat were selected from a standardized database (IAPS). This provides a reasonable guarantee that threatening pictures provide some degree of threat to the participant. However, this is only inferred in the context of our experiment: a methodologically more sound study to be performed in the future could include a manipulation check to determine the strength of threat the threatening (vs. neutral) pictures generate on a trial-by-trial basis. In addition, it has not been checked whether the participants had guessed the hypothesis of the study and this could be considered a limitation of the research. Another potential limitation of this study concerns the omission of disorganized attachment, which was not measured in this study. It was, in fact, found to be related to BMI and uncontrolled eating (Wilkinson et al., 2020).

## Conclusion

According to attachment theory, when under threat, seeking care and support are confirmed to be the primary strategy with which individuals regulate their negative emotions. However, the frequency of employment of this dominant strategy is associated with the attachment orientation of the specific individual. The differences that emerge in the threatening condition between anxiety and avoidance individuals demonstrate the different strategies of negative emotion regulation that the two styles entail. On one hand, because of their ambivalent mental representation of the caregiver as intermittently available, individuals with an anxious attachment tend to select strategies to cope with distress consisting of the choice of care less often than low-anxious individuals. On the other hand, because of their mental representations of the caregiver as unavailable, in neutral situations, without distress, the default mode of the response of avoidant individuals is defensive, but under threat and together with the representation of care and social support, they might opt to choose carefully as low-avoidant individuals.

Overall, our findings support the association between anxious attachment individuals and their greater difficulties with seeking support and contribute to the understanding of the conditions enabling different behavioral responses in the presence of avoidant attachment.

## Data Availability Statement

The raw data supporting the conclusions of this article will be made available by the authors, without undue reservation.

## Ethics Statement

The studies involving human participants were reviewed and approved by Ethics Committee of the University of Padova, Italy. The patients/participants provided their written informed consent to participate in this study.

## Author Contributions

AU: conceptualization, methodology, and writing—original draft, review, and editing. ME: investigation and formal analysis. CM: methodology and writing—review. All authors contributed to the article and approved the submitted version.

## Funding

The study was supported by an internal funding (2020) received from Sassari University, Italy and Sardinian L.R. n.7/2007 (2019).

## Conflict of Interest

The authors declare that the research was conducted in the absence of any commercial or financial relationships that could be construed as a potential conflict of interest.

## Publisher's Note

All claims expressed in this article are solely those of the authors and do not necessarily represent those of their affiliated organizations, or those of the publisher, the editors and the reviewers. Any product that may be evaluated in this article, or claim that may be made by its manufacturer, is not guaranteed or endorsed by the publisher.

## Footnotes

^1^Threatening pictures: 1120 – 1300 – 1304 – 1930 – 3230 – 3500 – 3530 – 6250 – 6312 – 6313 – 6315 – 6350 – 6370 – 6510 – 6540 – 6550 – 6560 – 6570 – 9414 - 9635.1. Neutral pictures: 7040 – 7002 – 7004 – 7018 – 7020 – 7026 – 7053 – 7059 – 7061 – 7062 – 7080 – 7090 – 7095 – 7150 – 7175 – 7185 – 7205 – 7211 – 7233 – 7235.

^2^Food pictures: 4 – 15 – 26 – 31 – 43 – 44 – 48 – 66 – 67 – 80 – 93 – 94 – 102 – 103 – 104 – 107 – 109 – 113 – 115 – 116 – 127 – 133 – 137 – 140 – 150 – 170 – 173 – 177 – 183 – 186 – 205 – 225 – 286 – 287 – 510 – 539 – 673 – 676 – 869 – 878.

^3^Care pictures: 1 – 2 – 3 – 4 – 5 – 8 – 9 – 11 – 12 – 14 – 15 – 16 – 19 – 20 – 22 – 23 – 24 – 25 – 26 – 27 – 28 – 29 – 30 – 32 – 34 – 35 – 40 – 42 – 46 – 47 – 48 – 49 – 50 – 51 – 52 – 55 – 56 – 57 – 59 – 64.
